# Carcinome papillaire sur kyste du tractus thyréoglosse: à propos de deux cas

**DOI:** 10.11604/pamj.2015.22.105.7006

**Published:** 2015-10-07

**Authors:** Lamia Dbab, Hassan Nouri, Nassim Iguelouane, Youssef Rochdi, Lahcen Aderdour, Abdelaziz Raji

**Affiliations:** 1 Service d'ORL et de Chirurgie Cervico-faciale, CHU Mohammed VI, Marrakech, Maroc

**Keywords:** Kyste du tractus thyréoglosse, carcinome papillaire, thyroïdectomie, Thyroglossal duct cyst, papillary carcinoma, thyroidectomy

## Abstract

L'objectif de ce travail est de présenté deux cas de carcinomes papillaire sur kystes du tractus thyréoglosse (KTT) et d'exposer notre attitude thérapeutique. Il s'agit de deux patientes, une âgée de 30 ans et l'autre de 45 ans, qui ont été opéré dans notre centre. Le diagnostic du carcinome papillaire sur KTT a été retenu après étude anatomopathologique du kyste après exérèse selon la technique de Sistrunk. Une thyroïdectomie totale suivie d'une irathérapie et d'une hormonothérapie freinatrice a été réalisé chez l'une des patientes, pour l'autre une thyroïdectomie totale associée à une hormonothérapie freinatrice a été préconisé. L'évolution été favorable après un recule de 4 ans pour la première patiente et de 2 ans pour la deuxième. La thyroïdectomie totale après exérèse complète du KTT est le traitement recommandé pour le carcinome papillaire sur KTT. Il a été démontré qu'un cancer latent de la thyroïde peut être développé même après des années après l'exérèse du KTT. Le traitement du carcinome papillaire sur KTT est bien codifié, permettant un excellent pronostic.

## Introduction

Les kystes du tractus thyréoglosse (KTT) sont des malformations cervicales congénitales, dues à une persistance anormale du canal thyréoglosse [1, 2]. Les localisations néoplasique au niveau du KTT sont rares, leur prévalence varie de 1 à 1,5% [1, 3, 4]. Nous rapportons deux observations médicales originales d'un carcinome papillaire sur KTT.

## Patient et observation

### Observation 1

**Figure 1:**
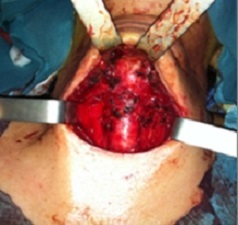
Site opératoire après thyroidectomie totale (Case 1)

Mme L.L âgée de 30 ans sans antécédents pathologiques particuliers, ayant consulté pour une tuméfaction cervicale antérieure augmentant progressivement de taille depuis l'enfance sans signes de compression mais devenant gênante sur le plan esthétique. L'examen clinique trouvait une patiente en bon état général avec une masse cervicale médiane ad-hyoïdienne, de consistance ferme, indolore, mesurant 2cm sur 2 cm, ascensionnant à la déglutition et à la protraction de la langue. Il y'avait pas de signes inflammatoires ni d'adénopathies cervicales. L'échographie montrait une masse cervicale antérieure mesurant 21 mm de diamètre, d'échostructure hétérogène renferment des débris hyperéchogènes faisant suspecter un KTT. La glande thyroïde était d'aspect normal sans adénopathies cervicales détectables. La patiente était opérée selon la technique de Sistrunk emportant en bloc le kyste, son cordon, le corps de l'os hyoïde et une collerette musculaire basilinguale. L'examen histopathologique de la pièce opératoire a révélé un microcarcinome papillaire sur KTT. Le taux de la thyroglobuline et de la TSH étaient normaux. Après une discussion multidisciplinaire, la patiente a bénéficié d'une thyroïdectomie totale (Figure 1), l'étude histopathologique a révélé un microcarcinome papillaire thyroïdien. Un balayage isotopique corps entier à l'iode 131 a révélé des résidus tumoraux thyroïdiens. Une ira thérapie a été prescrite, avec une hormonothérapie à dose freinatrice, l'évolution était favorable : absence de récidive après un recul quatre ans.

### Observation 2

**Figure 2:**
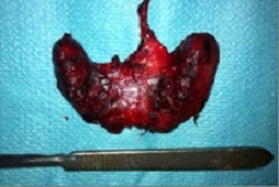
Pièce opératoire de la thyroidectomie totale (Case 2)

Mme M.F âgée de 45 ans sans antécédents pathologiques particuliers, qui s'est présentée pour une masse cervicale antérieure évoluant depuis 5 ans et augmentant progressivement de volume, indolore sans signes de compression. L'examen clinique à son admission retrouvait une patiente en bon état général, présentant une tuméfaction cervicale antérieure médiane, de consistance ferme non douloureuse, de 2cm sur 1cm, mobile à la déglutition et à la protraction de la langue, sans signes inflammatoires en regards sans adénopathies cervicales. L'échographie cervicale montrait une masse ad hyoïdienne de 22 mm de diamètre hétérogène avec des zones de kystisation faisant suspecter un KTT. La glande thyroïde était d'aspect normal et il n'y avait pas d'ADP cervicales. Une résection chirurgicale du KTT a été réalisée selon la technique de Sistrunk. L'étude anatomopathologique a montré une masse kystique avec à l'étude microscopique des anomalies cytonucléaires en faveur d'un carcinome papillaire. Après une discussion multidisciplinaire, la patiente a bénéficié ensuite d'une thyroïdectomie totale sans curage ganglionnaire vu l'absence d'adénopathies cervicales (Figure 2), l'examen anatomopathologique de la pièce de résection a objectivé une dystrophie thyroïdienne sans signes de malignité. L'évolution a été favorable: absence de récidive après deux ans.

## Discussion

Les kystes du tractus thyréoglosse (KTT) sont des malformations cervicales congénitales, dues à une persistance anormale du canal thyréoglosse reliquat embryonnaire de la migration de l'ébauche thyroïdienne depuis la base de la langue jusqu'à sa localisation anatomique définitive [1, 5]. Les localisations néoplasiques au niveau du tractus thyréoglosse sont rares. Leurs prévalences varient de 1 à 1,5% des kystes. Elles surviennent essentiellement chez l′adulte vers la quatrième décennie [1, 3, 6]. La première description de la cancérisation au sein du KTT remonte au 1911 avec BRENTANO [3]. Le type papillaire est le plus répandu, il est retrouvé dans environ 83 % des cas. Les autres types sont représentés par les carcinomes mixtes papillo-folliculaires dans 8% des cas, les carcinomes à cellules squameuses dans 6% des cas avec quelques cas de carcinomes à cellules de Hûrthle et de carcinomes folliculaires, anaplasiques et epidermoide. Aucun cas de carcinome médullaire n'a été décrit dans la littérature [1, 3, 6–8].

Beaucoup d'auteurs pensent que ces carcinomes se développent de novo au sein du KTT [9], son origine serait le tissu thyroïdien normal présent aussi bien dans la paroi du kyste que tout le long du trajet du tractus, et dont la fréquence varie de 1,5 à 62% des kystes [7, 8]. D'autres auteurs, croient que le canal thyréoglosse constitue une voie naturelle pour la propagation des carcinomes à partir de la glande thyroïde [10]. La présentation clinique est généralement similaire à celle d'un KTT simple, ce qui explique sa découverte le plus souvent fortuite à la suite d'un examen anatomopathologique de la pièce opératoire [1, 4]. Or, quelques signes cliniques doivent pousser le praticien à suspecter un éventuel processus néoplasique, en particulier le caractère dur, fixe et/ou irrégulier de la masse cervicale qui aurait augmenté rapidement progressive de taille ou s'associé à des adénopathies cervicales [3]. La conduite devant la découverte d′un cancer à l′examen anatomopathologique de la pièce d′exérèse d'un KTT est un sujet de controverse, notamment en ce qui concerne la nécessité ou non d'une thyroïdectomie associée à l'exérèse du KTT [6].

Selon plusieurs auteurs, il est recommandé de compléter le geste chirurgical initial par une thyroïdectomie totale. Les raisons invoquées sont la fréquence de l'association des KTT dégénérés avec des carcinomes primitifs de la thyroïde qui varie de 11 à 40% et la garantie d'un meilleure suivi, puisque dans une métanalyse, PATEL a montré que seule l'étendue du geste chirurgical initial constitue un variable significatif quant à la survie [1–3, 5, 6]. Cette thyroïdectomie serait plus indispensable en cas d'envahissement tumoral de la paroi du kyste, en cas d'individualisation d'un type histologique vésiculaire ou epidermoide ou de révélation clinique ou échographique d'une lésion nodulaire thyroïdienne et pour certains auteurs lorsqu'il existe un doute sur la capacité du patient à adhérer au suivi médical régulier [1]. Le curage ganglionnaire s'effectuerait en cas de présence d'adénopathies suspectes cliniquement ou à l'échographie [1–3]. Une scintigraphie du corps entier à l'iode 131 et un dosage de la thyroglobuline doivent être réalisée après thyroïdectomie. L'existence de résidus tumoraux à la scintigraphie imposera une irathérapie à dose ablative. L'hormonothérapie thyroïdienne à dose freinatrice est toujours indiquée [1, 2]. Des récidives peuvent survenir plusieurs années, voire des décennies plus tard, elles sont souvent astreignantes, d'où la nécessité d'une surveillance efficace à vie [7]. D′autres comme GEOK ne voient pas l'intérêt de la thyroïdectomie si la thyroïde est indemne [10]. Leurs arguments sont l′augmentation de la morbidité du fait d'interventions itératives, la possibilité de réaliser un suivi efficace avec ré-intervention dans un second temps en cas de découverte d'un cancer thyroïdien, et en fin le bon pronostic de ces cancers. Le pronostic des carcinomes sur KTT semble être meilleur que celui de la thyroïde du fait de la rareté des métastases à distance [1, 3]. La survie à 5 et à 10 ans est estimée respectivement à 100 et à 95,6% selon PATEL [3, 10].

## Conclusion

Les auteurs ajoutent à la littérature deux cas de carcinomes papillaire sur KTT, cette pathologie est rare, le diagnostic repose sur l'étude anathomopathologique, et le traitemet est actuellement bien codifié ceci pour permettre d'améliorer le pronostic.
